# The Putzig partners DREF, TRF2 and KEN are involved in the regulation of the *Drosophila* telomere retrotransposons, *HeT-A* and *TART*

**DOI:** 10.1186/1759-8753-4-18

**Published:** 2013-07-03

**Authors:** Rute Silva-Sousa, Míriam Díaz Varela, Elena Casacuberta

**Affiliations:** 1 Institute of Evolutionary Biology (CSIC-Universitat Pompeu Fabra), Passeig de la Barceloneta, 37-49, Barcelona 08003, Spain

## Abstract

**Background:**

Telomere maintenance in *Drosophila* relies on the targeted transposition of three very special non-LTR retrotransposons, *HeT-A, TART*, and *TAHRE* (HTT)*.* The sequences of the retrotransposon array build up the telomere chromatin in this organism. We have recently reported the role of the chromosomal protein Putzig/Z4 in maintaining a proper chromatin structure at the telomere domain of *Drosophila*. Because the Putzig protein has been found in different cellular complexes related with cell proliferation, development, and immunity, we decided to investigate whether the previously described Putzig partners, DREF/TRF2 and KEN, could also be involved in the telomere function in this organism.

**Results:**

We have found that mutant alleles for *Dref/Trf2* and *Ken* show alterations in *HeT-A* and *TART* expression, suggesting a possible role of these protein complexes in the regulation of the telomere retrotransposons. In agreement, both *HeT-A* and *TART* contain the specific DNA binding sequences for the DREF and the KEN protein proteins.

**Conclusions:**

We have identified three new negative regulators involved in the control of the expression of the telomeric retrotransposons, *Dref, Trf2*, and *Ken*. Our results offer some clues on which other chromatin-related proteins might be involved in telomere regulation and retrotransposon control.

## Background

The telomeres in *Drosophila* are constituted by an array of three specialized non-LTR retrotransposons, *HeT-A*, *TART*, and *TAHRE* (HTT array), whose targeted transpositions at the end of the chromosomes are analogous to the telomere replication performed by the holoenzyme telomerase in most eukaryotes [1-3]. Since the main genes involved in telomere elongation in *Drosophila* are embedded at the telomere chromatin, a study of the regulation and structure of the chromatin at this domain is important in understanding the telomere function in this organism. The chromatin at the telomere domain, the HTT array, attracts a different set of proteins from the subtelomeric domain, telomere associated sequences (TAS), and nucleates a specific class of chromatin with mixed characteristics of heterochromatin and euchromatin [4-6], and RSS, unpublished observations).

The chromosomal protein Z4/Putzig (Pzg) is a seven zinc-finger protein known to localize at polytene chromosome interbands and necessary to maintain the band-interband structure in these chromosomes [7]. A study using a *Drosophila* mutant line, *tel1* characterized by the presence of telomeres ten times longer than the average wild-type *Drosophila* telomeres [8], identified Pzg as a component of the telomere domain [4]. These findings led us to investigate the role of Pzg at *Drosophila* telomeres. We found that the lack of Pzg disturbs the structure of the telomeric chromatin affecting the stability of the telomeres and causing telomere fusions (TFs) [6]. The telomere function of Pzg is coordinated with other proteins present at the HTT array, such as JIL-1 or HP1a [6]. The equilibrium between these proteins is one of the keys to obtaining a precise level of expression of the telomere retrotransposons, *HeT-A* and *TART*. A recent study has also confirmed the presence of Pzg at the telomeres when screening for proteins that interact with another component of the HTT array, the Prod protein [9,10].

Pzg is not a telomere-specific protein and has been shown to be an important cofactor in at least three different pathways related with chromatin remodeling. In most cases, Pzg exerts its effects by mediating chromatin changes and acts as an activator; these are the cases of the nucleosome remodeling factor (NURF) and the DREF/TRF2 complexes [11,12]. The role of Pzg in the DREF/TRF2 complex is related with the necessary remodeling of the chromatin around the promoters of replication-related genes. The DREF homo-dimer binds specifically to the DRE sequence and, together with TRF2, is required for the cellular shift from the resting state into the proliferating state [13]. Nevertheless, Pzg can also negatively regulate the expression, as, for example, when it directly binds the co-repressor KEN in the JAK/STAT pathway [14]. The identification of Pzg in a protein complex composed of KEN and NURF in immunoprecipitation experiments, together with the observation of melanotic tumors in *pzg* mutant flies, which was due to an overexpression of defense response genes, strongly suggested the involvement of Pzg and NURF in the transcriptional repression of the JAK/STAT pathway genes [14,15].

Understanding whether any of these mechanisms involving Pzg could be linked to its telomere role is relevant to a better understanding of both telomere biology in *Drosophila* and how the regulation of the non-LTR retrotransposons *HeT-A* and *TART* could be related to the replication or defense mechanism of the organism.

## Results

### Mutations in *Dref*, *Trf2*, and *Ken* affect the telomeric retrotransposons *HeT-A* and *TART*

We investigated whether mutations in *Dref*, *Trf2*, and *Ken* affected the expression of the telomeric retrotransposons *HeT-A* and *TART*. As mentioned, Pzg has been found in the same protein complexes as DREF and KEN. TRF2 (TATA-box-binding protein (TBP) related factor 2) has been found in a complex with NURF and DREF. It was demonstrated that the recognition of promoters of replication-related genes by TRF2 depended on the presence of DREF that directly binds to specific DNA motifs [13]. If the function of Pzg at the telomeres depends on the action of the DREF complex, we would expect similar effects of mutant alleles of *Trf2* and *Dref* over the telomere retrotransposons. Therefore, the mutant alleles included in this study are *Dref*^*KG0994*^, *Trf*^*260071*^, and *Ken*^*1*^ mutants.

We have previously observed that null mutants of *pzg* do not affect the expression of the *HeT-A* retrotransposon [6], although the hypomorph mutant *Z4*^*7.1*^ affects its expression and genomic copy number [6]. We included two different *pzg* mutant alleles in these studies, the *pzg* hypomorph (*Z4*^*7.1*^) and a *pzg* null mutant (*pzg*^*66*^), in order to investigate whether they affected the expression of the *TART* retrotransposon. For all mutants, we analyzed the levels of *HeT-A* and *TART* mRNA by quantitative real-time PCR. Because the number of copies of the telomeric retrotransposons varies among stocks, we normalized the *HeT-A* and *TART* mRNA data by the number of copies of the retrotransposon in each stock, to obtain the level of expression of each copy of *HeT-A* and *TART*. Moreover, the presence of a higher copy number of one of the telomere retrotransposons in a mutant allele could be indicative of an increased expression and rate of terminal transposition.

**Figure 1:**
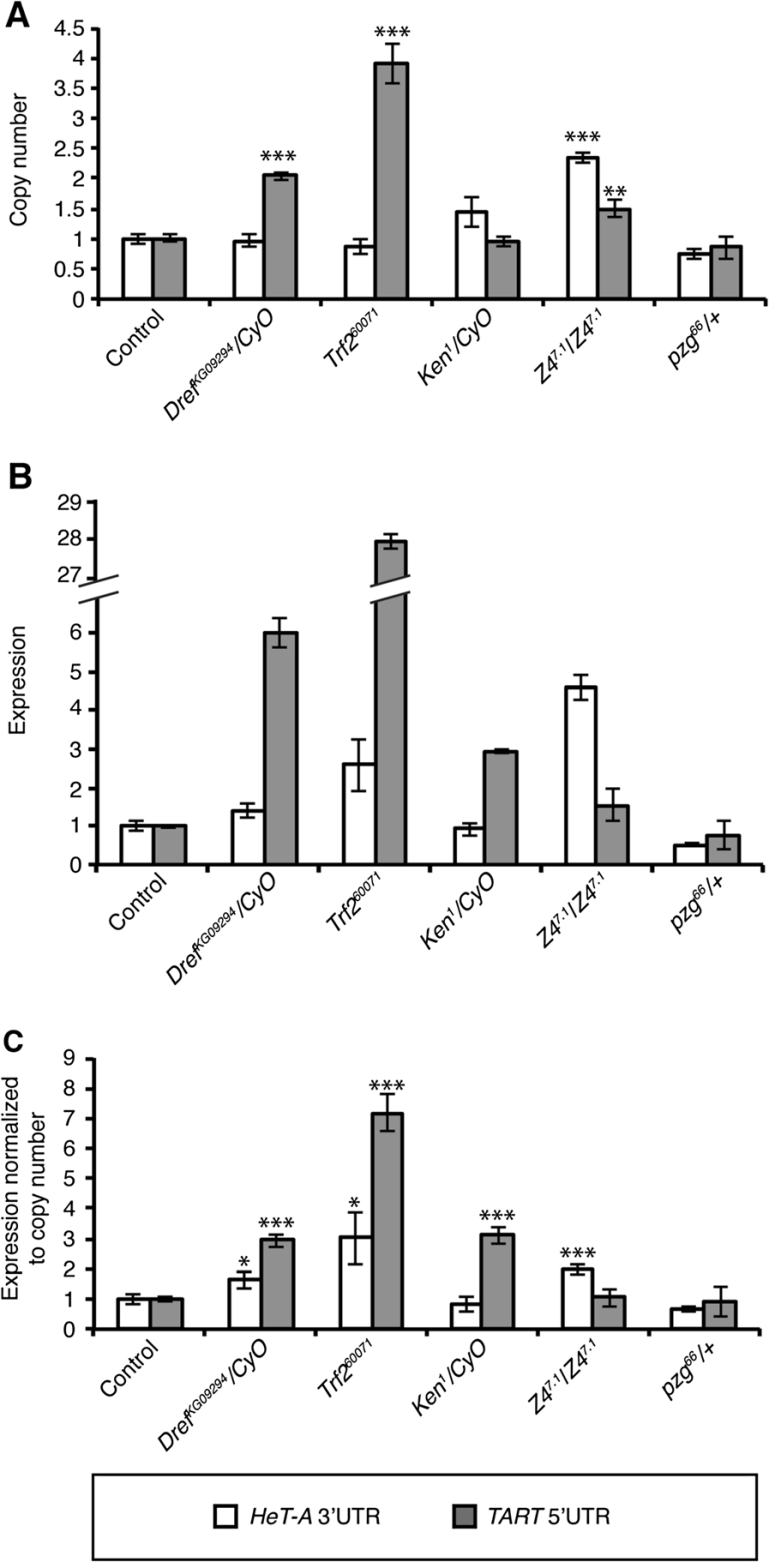
***HeT-A *and *TART *expression and copy number in *Dref*, *Trf2*, *Ken*, and *pzg *mutants. (A)***Dref* and *Trf2* mutants have more *TART* copies than control flies but no difference in *HeT-A* copies is observed. *Ken* and *pzg*^*66*^/+ mutants do not affect *HeT-A* or *TART* copy number. *Z4*^*7.1*^/*Z4*^*7.1*^ mutants have more *HeT-A* and *TART* copies. **(B,C)***HeT-A* transcripts increase in *Dref*, *Trf2*, and *Z4*^*7.1*^/*Z4*^*7.1*^ mutants but no effect is observed in *Ken* mutants. *TART* transcripts increase in *Dref*, *Trf2,* and *Ken* mutants and do not change in *pzg* mutants. *HeT-A* is represented in white bars and *TART* in grey bars. Error bars represent standard deviations of three independent experiments. Asterisks indicate statistically significant differences (* *P* < 0.05 to 0.01; ** *P* < 0.01 to 0.001; ***, *P* < 0.001) in *HeT-A* and *TART* expression and copy number of each mutant compared with respective controls. For all the analyzed stocks, the number of copies and level of expression were normalized to their respective controls, *w*^*1118*^ and *ry*^*506*^, which in turn were normalized to 1, to simplify the interpretation of the data.

To determine the number of copies of *HeT-A* and *TART* in each stock, we extracted genomic DNA from third-instar larvae without salivary glands. *Dref*^*KG0994*^, *Trf*^*260071*^, and *Ken*^*1*^ mutant alleles did not show differences in *HeT-A* copy number (Figure 1A), but in the *Z4*^*7.1*^/*Z4*^*7.1*^ hypomorph mutant, an increase in *HeT-A* copy number was observed, as we have previously demonstrated [6]. Results for the *TART* element are different; *Dref*^*KG0994*^, *Trf*^*26007*^,^*1*^ and the *Z4*^*7.1*^/*Z4*^*7.1*^ alleles show a significant increase of *TART* copies in their genomes, while *Ken*^*1*^ and the null allele of *pzg*, *pzg*^*66*^, do not show a significant change in the number of copies of *TART* in their genomes (Figure 1A).

Next, we analyzed the mRNA levels of *HeT-A* and *TART* in the same mutants. To obtain the expression data we extracted mRNA from whole third-instar larvae and analyzed them by quantitative real-time PCR. Unlike *HeT-A*, the *TART* element does not show an increase in transcription in any of the *pzg* mutant alleles after normalizing the data. On the other hand, in accordance with the genomic copy number obtained, a significant increase in *TART* expression was observed for the *Dref*^*KG0994*^ and the *Trf*^*260071*^ alleles. These same alleles show a similar behavior for the expression of the *HeT-A* retrotransposon although less accentuated than for its telomere partner (Figure 1B,C). These observations are in accordance with a possible link between the role of Pzg and the DREF/TRF2 protein complexes in the control of the telomere retrotransposons. Finally, the *Ken*^*1*^ allele also shows an increase in *TART* expression although no effect in *HeT-A* expression was observed (Figure 1B,C). These results indicate that *Dref*^*KG0994*^, *Trf*^*260071*^, and *Ken*^*1*^ mutant alleles affect gene expression differently at the telomere in *Drosophila*.

### The *TART* promoter contains DREF binding sequences

**Figure 2:**
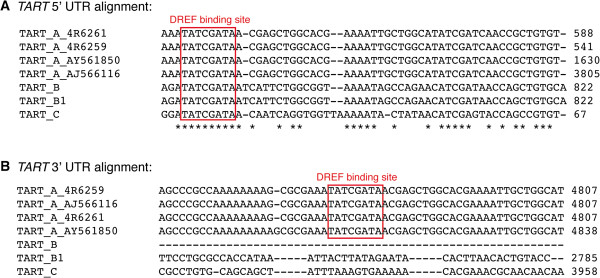
**DREF binding sites.** Nucleotide sequence alignment of *TART A*, *B*, and *C* subfamilies using ClustalW software. **(A)***TART* 5′ UTR alignment; DREF binding site is 100% conserved in all *TART* subfamilies. **(B)***TART* 3′ UTR alignment; DREF binding site is only conserved in the *TART A* subfamily. The alignment was performed for each domain, 5′ UTR, open reading frame, and 3′ UTR, but only the region corresponding to the DREF binding site is shown. Numbers on the right of the alignment refer to the position corresponding to each sequence.

After showing that the DREF/TRF2 complex had a role in controlling the expression of the telomeric retrotransposons, *HeT-A*, *TART*, and *TAHRE*, we searched for the presence of the DREF binding sequence (5′-TATCGATA) along the sequence of the telomeric retrotransposons [16]. We did not find the DREF motif in the sequence of *HeT-A* and *TAHRE*. However, we were able to identify two DREF binding sequences in *TART*; one in the 5′ UTR around 170 bp downstream of the transcription start site and another around 600 bp upstream the end of the 3′ UTR. To investigate whether these binding sites were conserved among the different *TART* subfamilies we performed a nucleotide sequence alignment using ClustalW software with the available copies in the databases (Figure 2). The sequence alignment revealed that the DREF binding site at the 5′ UTR was highly conserved among all *TART* subfamilies (Figure 2A) while the 3′ UTR sequence was only present in the *TART A* subfamily (Figure 2B). The DREF binding site at the *TART* 5′ UTR is located at the *TART* promoter that drives sense transcription, while the 3′ UTR binding site lies around the area where a putative *TART* anti-sense promoter has been proposed [17].

### *HeT-A*, *TART* and *TAHRE* contain KEN binding sites

**Figure 3:**
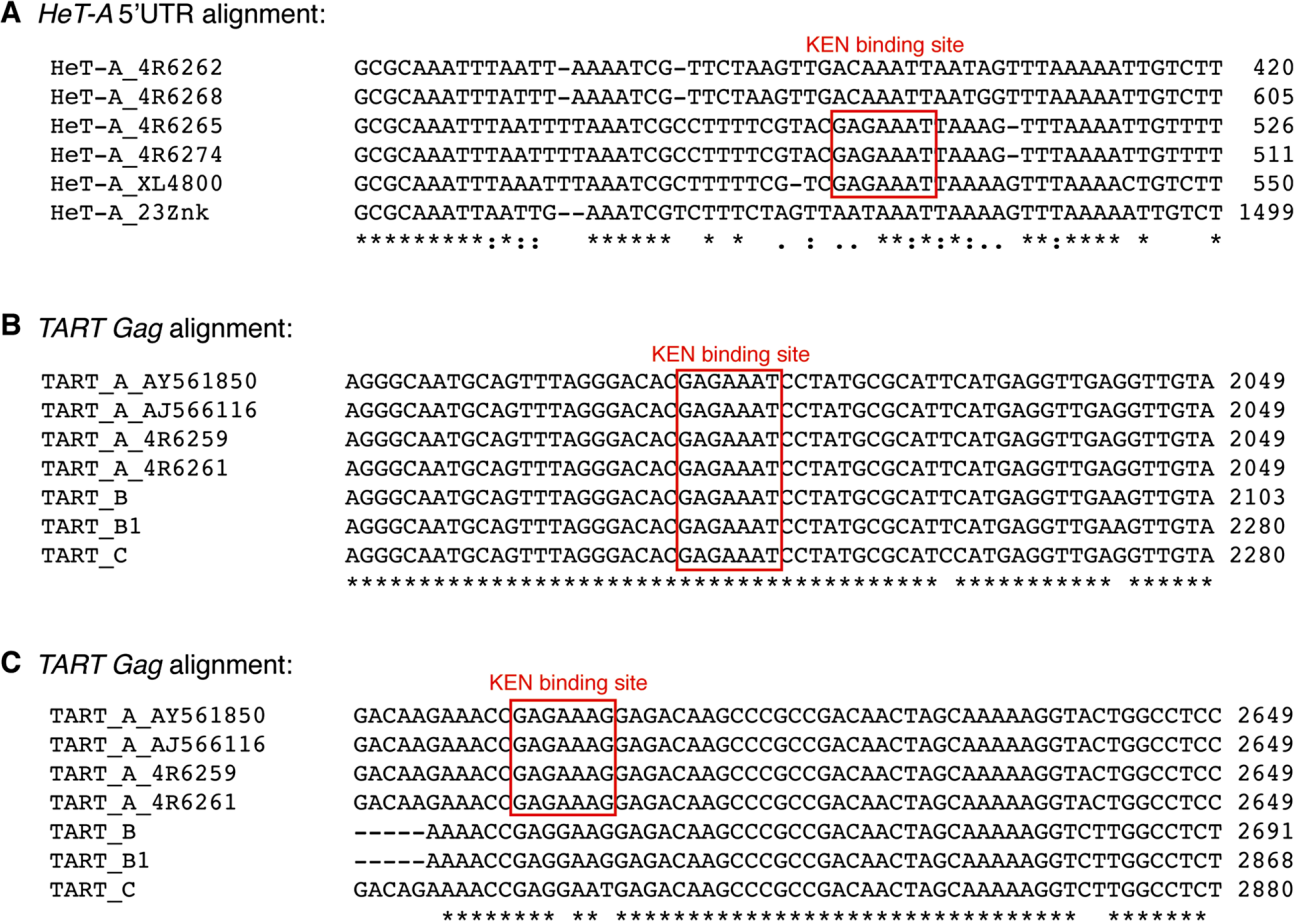
**KEN binding sites. (A)***HeT-A* 5′ UTR alignment; KEN binding site is conserved in three of the six *HeT-A* sequences analyzed. **(B)***TART Gag* alignment; KEN binding site is conserved in the three *TART* subfamilies. **(C)***TART Gag* alignment; the second KEN binding site is only conserved in the *TART A* subfamily. Nucleotide sequence alignment was performed using ClustalW software. The alignment was performed for each domain, 5′ UTR, open reading frame, and 3′ UTR, but only the region corresponding to the DREF binding site is shown. Numbers on the right of the alignment refer to the position corresponding to each sequence.

Similarly, we searched for KEN binding sites (5′-GAGAAAK, K = G/T) [18] in *HeT-A*, *TART*, and *TAHRE*. We found that this sequence was present in the three telomeric retrotransposons. *HeT-A* has a KEN binding site at the 5′ UTR that is conserved in three of the six analyzed sequences (Figure 3A). For this analysis, we only used the complete *HeT-A* sequences available in the databases (for more information, see [19]). *TART* has two KEN binding motifs inside the Gag coding sequence (ORF1); one is present in the three *TART* subfamilies, while the other is only present in the *TART A* subfamily (Figure 3B,C). Finally, in *TAHRE*, we found six copies of the *KEN binding site* (data not shown), three at the 5′ UTR, one at the reverse transcriptase domain (ORF 2), and two at the 3′ UTR. We could not perform a sequence multi-alignment analysis for *TAHRE*, since only one full sequence is available in databases [20]*.*

Both these observations, the alterations in the levels of expression of the telomere retrotransposons in mutant alleles of *Dref, Trf2*, and *Ken* and the presence of both DREF and KEN motifs in the sequences of the different telomere retrotransposons, suggest that the complexes that these proteins nucleate are able to bind to the telomeric array, and therefore that they are susceptible to regulate the expression of the telomeric retrotransposons. In future, *in vivo* evidence for these bindings will confirm the regulatory role of the DREF/TRF2 and Ken protein complexes at the telomere array.

## Discussion

We have previously demonstrated that the product encoded by the *Z4/putzig* gene is involved in maintaining telomere stability in *Drosophila*[6]. Because Pzg is not a telomere-specific protein and has been related with different protein complexes involved in diverse cellular functions, we decided to investigate whether the role of Pzg at the telomeres could involve any of the previously defined Pzg partners from other protein complexes [11,13-15].

### *Dref* and *Trf2* control the expression of *HeT-A* and *TART*

Although a previous study had reported that no DREF binding sites were present at the HTT array [10], we identified two DREF binding sites in the *TART* element of *D. melanogaster*; one at the 5′UTR, highly conserved among *TART* subfamilies, and another at the 3′UTR, only conserved in the *TART A* subfamily (Figure 2). The high conservation of the 5′UTR DREF binding site, suggests the existence of selective pressure acting at this DNA binding motif. Interestingly, the *TART A* subfamily, the one that contains both DREF binding motifs, is the more abundant of the *TART* subfamilies in *D. melanogaster*[21]. In addition to the presence of the DREF DNA binding motif in the sequence of the *TART* element, the recruitment of the DREF/TRF2 complex at the telomeres has been recently demonstrated [10].

We have observed an increase of transcription of both *HeT-A* and *TART* retrotransposons in the *Dref*^*KG09294*^ and *Trf*^*260071*^ mutant alleles (Figure 1C). In agreement with the presence of DREF binding sites in the *TART* element, we find that the difference in *TART* transcription for the *Dref*^*KG09294*^ and *Trf*^*260071*^ mutant alleles is especially significant. Moreover, both the *Dref*^*KG09294*^ and *Trf*^*260071*^ mutant alleles contain a higher copy number of *TART* elements, suggesting that this increase in transcription is translated in an increase in terminal transposition. In a *Dref*^*KG09294*^ and *Trf*^*260071*^ mutant background, the intermingled nature of *HeT-A* and *TART* at the HTT array [22] could help to spread the possible change in chromatin structure caused by the lack of binding of DREF/TRF2 at the *TART* sequences towards the *HeT-A* promoter, provoking an indirect increase in the transcription of *HeT-A* despite not containing DREF binding sites (Figure 1C).

The increase in expression from the HTT array in the mutant alleles of *Dref* and *Trf2* indicates that in a wild-type situation this protein complex behaves as a repressor of the telomere retrotransposons. *A priori*, we were expecting DREF/TRF2 to activate the telomere expression in synchrony with cell proliferation [11,13]. Nevertheless, in accordance with our results, the DREF/TRF2 complex has also been reported to contribute to gene repression.

The DREF/TRF2 complex competes for DNA binding with the BEAF (boundary element-associated factor) because both DREF and BEAF have overlapping binding sites (BEAF binds to 5′-CGATA motifs) [23]. Moreover, it was shown that both proteins are able to target the same promoter [24]. Boundary elements have the ability to insulate a transgene from its chromosomal context by blocking enhancer-promoter interactions and heterochromatin spreading [25]. In *D. melanogaster*, BEAF associates preferentially with active transcribed genes [26]. The presence of BEAF at DREF-regulated genes leads to a decrease in the deposition of the heterochromatic mark H3K9me3, de-repressing those genes from the surrounding heterochromatin [24,27,28]. Our results are compatible with this scenario, in which equilibrium between the binding of both complexes would be necessary to obtain a fine-tuned regulation of the expression of the telomere retrotransposons. The binding of BEAF to the DREF binding sites at the *TART* promoter would protect the telomere retrotransposon from a repressive environment, and the binding of DREF would protect the genome from an excessive transcription, transposition, and telomere elongation. In a DREF mutant background, a major occupancy of BEAF at the HTT array would create an opening of the surrounding chromatin and, consequently, also increase the levels of *HeT-A* expression.

Our initial hypothesis was that Pzg could be exerting its role at the telomeres through its interaction with the DREF/TRF2 complex. In contrast with the increase in *TART* transcription observed in the *Dref*^*KG09294*^ and *Trf*^*260071*^ mutant alleles, the results obtained with two *pzg* mutant alleles, *Z4*^*7.1*^ hypomorph and *pzg*^*66*^ null, show no change in the expression level of the *TART* retrotransposon. This result suggests that although the DREF/TRF2 has been related with Pzg in other situations, in this case they might be acting independently.

More chromatin modifiers have been related with the DREF/TRF2 complex, like the linker Histone H1, which is also involved in heterochromatin and transposable element gene expression as well as the NURF subunit ISWI (involved in ATP dependent nucleosome sliding) [29-32]. The loss of *Trf2* could also affect the telomere chromatin through a secondary effect on these other heterochromatin components. Future studies involving additional mutant alleles of the mentioned chromatin components will help to elucidate the molecular mechanism by which the DREF/TRF2 complex affects the telomeres.

### KEN is a repressor of *TART*

The JAK/STAT signaling pathway is responsible for the activation of the immune response genes [18,33]. KEN is a well-defined repressor of the JAK/STAT pathway and competes with STAT for the binding to a target gene [33]. Recently, NURF301 and Pzg were also found to be recruited by KEN and repress the JAK/STAT pathway [14,15]. When we analyzed the effect of the *Ken* mutant allele *Ken*^*1*^ in *Drosophila* telomeres, an increase in *TART* expression was observed but no effects on *HeT-A* expression were detected (Figure 1C). Accordingly, we found that the KEN binding sequences are not conserved in all the analyzed *HeT-A* sequences in opposition to the high conservation of at least one of the KEN binding sites in the *TART* sequence (Figure 3). A possible explanation could be that KEN might be involved in the recruitment of the chromatin-remodeling complex NURF to *TART* sequences repressing its transcription. In a *Ken* mutant background, the NURF complex is not recruited to the telomeres, leading to a relaxation of the chromatin. The fact that no effect on *HeT-A* expression is observed in *Ken* mutants suggests that the degree of chromatin relaxation is weaker than the one observed in *Dref* and *Trf2* mutants. As with the DREF/TRF2 complex, we have found that the *pzg* alleles here analyzed do not show a change in transcription equivalent to the one observed in the *Ken*^*1*^ mutant. This result suggests a possible independent role of *pzg* and the *Ken* at *Drosophila* telomeres. We do not know whether the role of *Ken* controlling the expression of the *TART* element keeps any relationship with the JAK/STAT pathway, but it is possible that independent roles of *Ken* from the JAK/STAT pathway exist and that the control of telomere transcription could be the first one to be described.

## Conclusions

We have identified three new genes involved in the regulation of the expression of the telomeric retrotransposons, *Dref, Trf2*, and *Ken*. Our results offer new insights in the composition and regulation of the telomere chromatin, pointing to unexpected relationships with other chromatin-related proteins and different pathways of the cell that had not been previously related with either telomere or retrotransposon regulation.

## Methods

### Fly stocks and crosses

Fly stocks were maintained and crosses preformed at 25°C on standard *Drosophila* corn meal medium. *w*^*1118*^ and *ry*^*506*^ were used as control, depending on the genetic background of each strain. *w*^*1118*^; *Trf*^*260071*^*/FM7c*, *ry*^*506*^; *Dref*^*KG09294*^*/CyO* and *ry*^*506*^; *Ken*^*1*^*/CyO* were obtained from Bloomington Stock Center. *w*^*1118*^; *Trf*^*260071*^*/FM7c* balancer was changed to *FM7c-GFP* to allow the selection of hemizygous males to perform the experiments. The three mutations correspond to P-element insertions inside the coding region. The hypomorph line *Z4*^*7.1*^*/TM3Sb*, lacking the promoter region*,* came from Harald Eggert and Harald Saumweber and the line *pzg*^*66*^*/TM6* was provided by Anja Nagel, this line is a *null* mutant that results in embryonic and early larval lethality.

### Sequence alignments

The sequence alignments were carried out using ClustalW software. For the accession number of the *HeT-A* sequences used in the alignments see [19] and for the accession number of *TART* and *TAHRE* sequences see [21].

### Genomic DNA extraction

Genomic DNA was extracted from adult flies to quantify the number of *HeT-A* and *TART* copies in each strain. Ten third-instar larvae without salivary glands were homogenized in 200 μl solution A (0.1 M Tris-HCl pH9.0, 0.1 M EDTA and 1% SDS) and incubated at 70°C for 30 min. 28 μl 8 M KAc were added and the samples incubated for 30 min on ice. Cell debris was harvested at maximum speed for 15 min at 4°C. The supernatant was transferred to a new tube and the DNA precipitated by adding 0.5 volumes isopropanol and centrifuging at 15.000 rpm for 5 min. Pelleted DNA was washed with 1 volume 70% ethanol and centrifuged. Finally, the DNA pellet was air-dried, and re-suspended in 50 μl 1× TE by rotating overnight at 4°C. After genomic DNA extraction, the number of copies was determined by quantitative real-time PCR using 2 ng of DNA per reaction.

Because certain endoreplication of the telomeric sequences exists in salivary glands, we depleted the samples from this tissue in order to have a copy number that would reflect the real copy number of the adult organism.

Primers used for *TART* amplification were, for TART_5′ UTR_F, GATAATAGCCGATAAGCCCGCCA, and for TART_5′ UTR_R, AAGACACAGCGGTTGATCGATATG. Primers used for *HeT-A* amplification were HeT-A_3′ UTR_F (CCCCGCCAGAAGGACGGA) and HeT-A_3′ UTR_R (TGTTGCAAGTGGCGCGCA). Primers used for *actin* amplification were Actin_F (GCGCCCTTACTCTTTCACCA) and Actin_R (ATGTCACGGACGATTTCACG).

### RNA extraction and cDNA synthesis

Total RNA was isolated from ten whole third-instar larvae and extracted using RNeasy Mini Kit (Qiagen) according to the manufacturer’s protocol. RNase Free DNase Set (Qiagen) was used to remove genomic DNA contaminations as follows: one on column during the extraction accordingly to manufacturer’s protocol, and two in solution for 2 hours at 37°C. RNA was cleaned by precipitation and its quality assessed using NanoDrop spectrophotometry.

One microgram of RNA was reverse transcribed into cDNA using Transcriptor First Strand cDNA Synthesis Kit (Roche) with oligo(dT) primers, and the expression of the different transcripts analyzed by quantitative real-time PCR. For each fly strain, two independent RNA extractions were prepared and analyzed independently three times. Primers used for *TART* amplification were, for TART_5′ UTR_F, GATAATAGCCGATAAGCCCGCCA, and for TART_5′ UTR_R, AAGACACAGCGGTTGATCGATATG. Primers used for *HeT-A* amplification were HeT-A_3′ UTR_F (CCCCGCCAGAAGGACGGA) and HeT-A_3′ UTR_R (TGTTGCAAGTGGCGCGCA). Primers used for *actin* amplification were Actin_F (GCGCCCTTACTCTTTCACCA) and Actin_R (ATGTCACGGACGATTTCACG).

We did not deplete the samples of salivary glands in the expression analysis, since the telomere retrotransposons are not expressed in this tissue [34].

### Quantitative real-time PCR

Quantitative Real Time-PCR was performed to determine *HeT-A* and *TART* copy number and expression. The iQ5 Multicolor Real-Time PCR Detection System was used and the iQ™ SYBR^®^ Green Supermix (BioRad) was used to prepare the reactions. Relative levels of *HeT-A* and *TART* expression were determined using the threshold cycle and normalized to actin levels. Three independent experiments of two samples each strain were performed.

## Abbreviations

BEAF: Boundary element-associated factor; DREF: DNA replication-related element-binding factor; HP1a: Heterochromatin protein 1a; HTT: HeT-A, TART and TAHRE array; LTR: Long terminal repeats; NURF: Nucleosome remodeling factor; PCR: Polymerase chain reaction; TAS: Telomere associated sequences; TBP: TATA-box-binding protein; TF: Telomere fusion; TRF2: TATA-box-binding protein related factor 2.

## Competing interests

The authors declare no competing interests.

## Authors’ contributions

RS-S participated in the design of the study, performed research, analyzed data, and helped in drafting the manuscript. MDV performed research and analyzed data. EC conceived the study, analyzed data, and wrote the manuscript. All authors approved the final manuscript.

## References

[B1] Silva-Sousa R López-Panadѐs E Casacuberta E Drosophila telomeres: an example of co-evolution with transposable elements Genome Dyn 2010 7 46 67 22759813 10.1159/000337127

[B2] Pardue ML DeBaryshe PG Drosophila telomeres: a variation on the telomerase theme Fly (Austin) 2008 2 101 110 18820466 10.4161/fly.6393

[B3] Capkova Frydrychova R Biessmann H Mason JM Regulation of telomere length in Drosophila Cytogenet Genome Res 2008 122 356 364 19188706 10.1159/000167823 PMC2637470

[B4] Andreyeva EN Belyaeva ES Semeshin VF Pokholkova GV Zhimulev IF Three distinct chromatin domains in telomere ends of polytene chromosomes in Drosophila melanogaster Tel mutants J Cell Sci 2005 118 5465 5477 16278293 10.1242/jcs.02654

[B5] Biessmann H Prasad S Semeshin VF Andreyeva EN Nguyen Q Walter MF Mason JM Two distinct domains in Drosophila melanogaster telomeres Genetics 2005 171 1767 1777 16143601 10.1534/genetics.105.048827 PMC1382029

[B6] Silva-Sousa R López-Panadès E Piñeyro D Casacuberta E The chromosomal proteins JIL-1 and Z4/Putzig regulate the telomeric chromatin in Drosophila melanogaster PLoS Genet 2012 8 e1003153 23271984 10.1371/journal.pgen.1003153 PMC3521665

[B7] Eggert H Gortchakov A Saumweber H Identification of the Drosophila interband-specific protein Z4 as a DNA-binding zinc-finger protein determining chromosomal structure J Cell Sci 2004 117 4253 4264 15292401 10.1242/jcs.01292

[B8] Siriaco GM Cenci G Haoudi A Champion LE Zhou C Gatti M Mason JM Telomere elongation (Tel), a new mutation in Drosophila melanogaster that produces long telomeres Genetics 2002 160 235 245 11805059 10.1093/genetics/160.1.235 PMC1461955

[B9] Török T Benitez C Takács S Biessmann H The protein encoded by the gene proliferation disrupter (prod) is associated with the telomeric retrotransposon array in Drosophila melanogaster Chromosoma 2007 116 185 195 17186256 10.1007/s00412-006-0090-4

[B10] Takács S Biessmann H Reddy HM Mason JM Török T Protein interactions on telomeric retrotransposons in Drosophila Int J Biol Sci 2012 8 1055 1061 22949888 10.7150/ijbs.4460 PMC3432853

[B11] Kugler SJ Nagel AC Putzig is required for cell proliferation and regulates Notch activity in Drosophila Mol Biol Cell 2007 18 3733 3740 17634285 10.1091/mbc.E07-03-0263 PMC1995712

[B12] Kugler SJ Nagel AC A novel Pzg-NURF complex regulates Notch target gene activity Mol Biol Cell 2010 21 3443 3448 20685964 10.1091/mbc.E10-03-0212 PMC2947479

[B13] Hochheimer A Zhou S Zheng S Holmes MC Tjian R TRF2 associates with DREF and directs promoter-selective gene expression in Drosophila Nature 2002 420 439 445 12459787 10.1038/nature01167

[B14] Kugler SJ Gehring EM Wallkamm V Krüger V Nagel AC The Putzig-NURF nucleosome remodeling complex is required for ecdysone receptor signaling and innate immunity in Drosophila melanogaster Genetics 2011 188 127 139 21385730 10.1534/genetics.111.127795 PMC3120143

[B15] Kwon SY Xiao H Glover BP Tjian R Wu C Badenhorst P The nucleosome remodeling factor (NURF) regulates genes involved in Drosophila innate immunity Dev Biol 2008 316 538 547 18334252 10.1016/j.ydbio.2008.01.033

[B16] Matsukage A Hirose F Yoo MA Yamaguchi M The DRE/DREF transcriptional regulatory system: a master key for cell proliferation Biochim Biophys Acta 2008 1779 81 89 18155677 10.1016/j.bbagrm.2007.11.011

[B17] Maxwell PH Belote JM Levis RW Identification of multiple transcription initiation, polyadenylation, and splice sites in the Drosophila melanogaster TART family of telomeric retrotransposons Nucleic Acids Res 2006 34 5498 5507 17020919 10.1093/nar/gkl709 PMC1636488

[B18] Arbouzova NI Bach EA Zeidler MP Ken & barbie selectively regulates the expression of a subset of Jak/STAT pathway target genes Curr Biol 2006 16 80 88 16401426 10.1016/j.cub.2005.11.033

[B19] Piñeyro D López-Panadès E Lucena-Pérez M Casacuberta E Transcriptional analysis of the HeT-A retrotransposon in mutant and wild type stocks reveals high sequence variability at Drosophila telomeres and other unusual features BMC Genomics 2011 12 573 22111838 10.1186/1471-2164-12-573 PMC3235214

[B20] Abad JP De Pablos B Osoegawa K De Jong PJ Martín-Gallardo A Villasante A TAHRE, a novel telomeric retrotransposon from Drosophila melanogaster, reveals the origin of Drosophila telomeres Mol Biol Evol 2004 21 1620 1624 15175413 10.1093/molbev/msh180

[B21] George JA DeBaryshe PG Traverse KL Celniker SE Pardue ML Genomic organization of the Drosophila telomere retrotransposable elements Genome Res 2006 16 1231 1240 16963706 10.1101/gr.5348806 PMC1581432

[B22] Pardue ML DeBaryshe PG Retrotransposons provide an evolutionarily robust non-telomerase mechanism to maintain telomeres Annu Rev Genet 2003 37 485 511 14616071 10.1146/annurev.genet.38.072902.093115

[B23] Hart CM Cuvier O Laemmli UK Evidence for an antagonistic relationship between the boundary element-associated factor BEAF and the transcription factor DREF Chromosoma 1999 108 375 383 10591997 10.1007/s004120050389

[B24] Emberly E Blattes R Schuettengruber B Hennion M Jiang N Hart CM Käs E Cuvier O BEAF regulates cell-cycle genes through the controlled deposition of H3K9 methylation marks into its conserved dual-core binding sites PLoS Biol 2008 6 2896 2910 19108610 10.1371/journal.pbio.0060327 PMC2605929

[B25] Barkess G West AG Chromatin insulator elements: establishing barriers to set heterochromatin boundaries Epigenomics 2012 4 67 80 22332659 10.2217/epi.11.112

[B26] Vogelmann J Valeri A Guillou E Cuvier O Nollmann M Roles of chromatin insulator proteins in higher-order chromatin organization and transcription regulation Nucleus 2011 2 358 369 21983085 10.4161/nucl.2.5.17860 PMC3796873

[B27] Hart CM Zhao K Laemmli UK The scs′ boundary element: characterization of boundary element-associated factors Mol Cell Biol 1997 17 999 1009 9001253 10.1128/mcb.17.2.999 PMC231825

[B28] Cuvier O Hart CM Käs E Laemmli UK Identification of a multicopy chromatin boundary element at the borders of silenced chromosomal domains Chromosoma 2002 110 519 531 12068969 10.1007/s00412-001-0181-1

[B29] Bouazoune K Brehm A ATP-dependent chromatin remodeling complexes in Drosophila Chromosome Res 2006 14 433 449 16821138 10.1007/s10577-006-1067-0

[B30] Siriaco G Deuring R Chioda M Becker PB Tamkun JW Drosophila ISWI regulates the association of histone H1 with interphase chromosomes in vivo Genetics 2009 182 661 669 19380479 10.1534/genetics.109.102053 PMC2710149

[B31] Lu X Wontakal SN Emelyanov AV Morcillo P Konev AY Fyodorov DV Skoultchi AI Linker histone H1 is essential for Drosophila development, the establishment of pericentric heterochromatin, and a normal polytene chromosome structure Genes Dev 2009 23 452 465 19196654 10.1101/gad.1749309 PMC2648648

[B32] Vujatovic O Zaragoza K Vaquero A Reina O Bernués J Azorín F Drosophila melanogaster linker histone dH1 is required for transposon silencing and to preserve genome integrity Nucleic Acids Res 2012 40 5402 5414 22406835 10.1093/nar/gks224 PMC3384340

[B33] Hombría JC Sotillos S JAK/STAT signalling: STAT cannot play with Ken and Barbie Curr Biol 2006 16 R98 R100 16461274 10.1016/j.cub.2006.01.021

[B34] George JA Pardue M-L The promoter of the Heterochromatic Drosophila Telomeric retrotransposon, *HeT-A*, is active when moved into euchromatic locations Genetics 2002 163 625 635 12618401 10.1093/genetics/163.2.625 PMC1462444

